# Unveiling Emerging Trends and Gaps in Scientific Research on Vertebrate Biodiversity in Tropical Savannahs

**DOI:** 10.1002/ece3.72917

**Published:** 2026-02-15

**Authors:** Marcelo Martins Ferreira, Paulo Estefano Dineli Bobrowiec, Karen Mustin, William Douglas Carvalho

**Affiliations:** ^1^ Programa de Pós‐Graduação em Ecologia Instituto Nacional de Pesquisas da Amazônia (INPA) Manaus Brazil; ^2^ Instituto Tecnológico Vale Belém PA Brazil; ^3^ Programa de Pós‐Graduação em Biodiversidade Tropical Universidade Federal do Amapá Macapá Brazil; ^4^ Department of Biodiversity, Ecology and Evolution Complutense University of Madrid Madrid Spain; ^5^ Terrestrial Ecology Group (TEG‐UAM), Department of Ecology, Faculty of Sciences Autonomous University of Madrid Madrid Spain; ^6^ Centro de Investigación en Biodiversidad y Cambio Global (CIBC‐UAM) Universidad Autónoma de Madrid Madrid Spain

**Keywords:** amphibians, birds, conservation, mammals, open ecosystems, reptiles

## Abstract

Savannahs are among the most threatened ecosystems in the world due to the rapid change in their land use for forestry, soybean cultivation, and pasture. However, savannahs are less studied than tropical forest ecosystems despite this intense anthropogenic pressure. As such, here we investigate the gaps and trends in scientific research on terrestrial vertebrates in tropical savannahs, via a systematic search for scientific articles on the Web of Science platform. Subsequently, to identify the geographic distribution of the studies, we divided the total number of articles by the area of the Savannah biome (in km^2^) that occurs in each country. Our results show that Africa has a deficit in scientific research on terrestrial vertebrates compared to Oceania and South America, and that this global trend in the distribution of studies is closely related to the Human Development Index. We also identified land use change and fire dynamics as the most studied drivers of biodiversity loss, while invasive species and climate change were the least well studied. Finally, our research revealed that about 80% of the articles focused on mammals and birds, and that phylogenetic and functional diversity were the least studied dimensions of vertebrate biodiversity in tropical savannahs. These results are concerning for conservation efforts, as they reveal not only a substantial geographic gap but also a limited and biased understanding of savannah biodiversity.

## Graphical abstract

Savannahs are among the most threatened ecosystems in the world due to the rapid change in their land use for forestry, soybean cultivation, and pasture. However, Africa has a deficit in scientific research on terrestrial vertebrates compared to Oceania and South America, and this global trend in the distribution of studies is closely related to the Human Development Index. Also, about 80% of the articles focused on mammals and birds, and phylogenetic and functional diversity were the least studied dimensions of vertebrate biodiversity in tropical Savannahs.

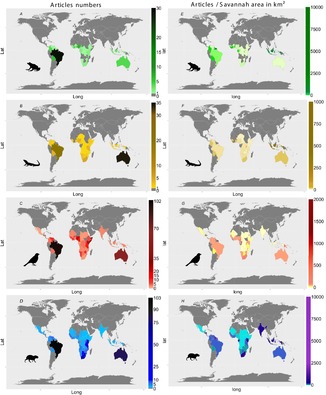

## 1 Introduction

Savannahs represent about 20% of all terrestrial ecosystems, making them one of the largest biomes in the Tropics (Scholes and Archer 1997), covering an area close to that of tropical forests (Savannah: 19,575,000 km^2^; Tropical forests: 24,348,000 km^2^; Murphy et al. 2016). The climate of the tropical Savannah region is characterized by well‐defined rainy and dry seasons, with average annual rainfall ranging from 1000 to 2500 mm (Staver et al. 2011). Tropical Savannahs are open ecosystems characterized by the occurrence of shrubby‐arboreal species with a predominance of herbaceous plants, especially those of the genera Poaceae and Cyperaceae, whose structure is strongly determined by the interaction between precipitation, fire, and dystrophic and sandy soils (Sankaran et al. 2005; Lloyd et al. 2008; Hoffmann et al. 2012; Lehmann et al. 2014; Scholes and Archer 1997; Staver et al. 2011). However, the magnitude of these factors varies substantially between continents. For example, in Africa and Australia, moisture availability and fire explain two to three times the variation in vegetation structure in Savannahs as compared with South America (Lehmann et al. 2014). The increased availability of moisture simultaneously promotes both an increase in plant biomass and in the frequency and intensity of fires (Archibald et al. 2013). Higher temperatures increase the photosynthetic rate of C4 grasses, generating greater plant biomass, and with warmer temperatures, all this biomass is more likely to dry out, facilitating fuel accumulation and increasing the intensity and frequency of fires in seasonally dry environments (Bond and Midgley 2000; Ripley et al. 2010), conditions widely found in the Savannahs of Africa and Australia (Lehmann et al. 2014). However, for the Savannahs of South America, acidic and infertile soils may be acting as constraints on both the distribution and structure of the vegetation (Staver et al. 2011; Lehmann et al. 2011). Thus, precipitation, fire, and soil conditions act as the main determinants of tree structure and as stabilizing mechanisms that sustain tropical Savannahs as predominantly open‐canopy, grassy ecosystems. Tropical Savannahs may also contain areas of seasonally flooded grasslands and islands of forest within the Savannah matrix (Bourliέre and Hadley 1970; Solbrig et al. 1996; Bond and Parr 2010; Ratnam et al. 2011; Scholes and Archer 1997).

The landscape heterogeneity of tropical Savannahs means that this biome is globally recognized for its high species richness and endemism, in addition to being home to many endangered species (Barbosa et al. 2007; Murphy et al. 2016; Carvalho and Mustin 2017; Pennington et al. 2018). The vertebrate biodiversity of tropical Savannahs is comparable to that of tropical forests (Pennington et al. 2018). For example, globally, Savannahs have an average richness of 78 mammal and 284 bird species per 10 km^2^, compared with an average richness of 87 mammal and 296 bird species per 10 km^2^ in tropical forests (Murphy et al. 2016). Furthermore, this biome is essential for maintaining many of the ecosystem services essential to humanity, such as water capture and the preservation of streams, rivers, and water tables, in addition to carbon sequestration (Greiner et al. 2009; Pennington et al. 2018).

Despite their importance in terms of biodiversity and ecosystem services, tropical Savannahs are among the most threatened ecosystems in the world, mainly due to land use changes, which have intensified in recent decades (Williams et al. 2022). Among tropical ecosystems, Savannahs have shown the greatest losses of forest cover (Hansen et al. 2013); between 2000 and 2013, approximately 11% of their original area was lost (~655.000 km^2^), which is equivalent to the area of France (Williams et al. 2020). For example, since the middle of the last century, the Savannahs of the Piedmont Llanos in Colombia and the Brazilian Cerrado have lost about 86% and 50% of their original area, respectively, mainly due to the conversion of natural vegetation into agricultural areas (Williams et al. 2020). This conversion includes soybean, corn and rice crop fields, as well as sugar cane, eucalyptus and acacia plantations, and the creation of pastures for livestock (Aragão et al. 2022; Gomes et al. 2019; Klink and Machado 2005; Mustin et al. 2017; Sano et al. 2010; Tovar et al. 2023). In addition to the direct effects of land use and land cover changes, the conversion of natural Savannah landscapes has significantly reduced precipitation and relative humidity, which has led to more fires (Hoffmann et al. 2002; Hoffman and Vogel 2008; Grégoire et al. 2012; Spera et al. 2016). All these anthropogenic actions have led to severe declines in biodiversity in Savannah ecosystems globally (Woinarski et al. 2011; Scholte et al. 2022; Xavier et al. 2026a, 2026b).

Despite alarming habitat losses, Savannahs remain poorly protected (Murphy et al. 2016; Pennington et al. 2018). In the Pantropical region, approximately 13% of the Savannahs are in areas designated for protecting and conserving biodiversity (Murphy et al. 2016). However, less than 4% of these areas remain intact (Murphy et al. 2016), while tropical forests have 25% of their extensions in protected areas (Murphy et al. 2016; Watson et al. 2016; Williams et al. 2022). In addition to the disparity in protection efforts, there are significant biases in scientific research, with most studies focusing on forest ecosystems and little attention paid to the factors driving biodiversity loss in Savannahs (Murphy et al. 2016; Pennington et al. 2018; Williams et al. 2022). In this context, systematic reviews are essential to identify trends and biases, contributing to the definition of new directions in research that aim to expand knowledge and strengthen conservation efforts for Savannah species, ecosystems, and habitats (Scarpelli et al. 2020; Llorente‐Culebras et al. 2023).

One of the main biases detected in global biodiversity reviews is the taxonomic group studied, which has relevant implications for species conservation. Birds and mammals are among the most extensively studied vertebrate groups (e.g., Llorente‐Culebras et al. 2023) and, consequently, have received greater attention in conservation policies, resulting in broader representation within management plans (García‐Macía et al. 2021). In general, the number of published studies on biodiversity varies between global regions, being closely related to the level of development, the per capita income of each country or region, and funding for scientific research, being greater in richer countries and the Global North (Fragoso 2024; Llorente‐Culebras et al. 2023; Titley et al. 2017). However, even in the most developed regions, most studies published on biodiversity over time have focused on the taxonomic dimension. Ignoring the functional (functional characteristics shared among the community) and phylogenetic (evolutionary history shared by a set of species) diversity of biodiversity means losing robustness in the search for environmental solutions (Cianciaruso et al. 2009).

Here, we focus on terrestrial vertebrates in tropical Savannahs and thus investigate the scientific knowledge about amphibians, reptiles, birds, and mammals worldwide through a systematic review of the available information in the literature. Specifically, we (i) identify which terrestrial vertebrate groups (amphibians, reptiles, birds, and mammals) have been most studied in tropical Savannahs, and (ii) analyze the distribution of studies across geographic regions. Furthermore, we (iii) investigate the relationship between the number of published studies and each country's Human Development Index (HDI), and (iv) identify which diversity metrics are most used to measure biodiversity (e.g., taxonomic, functional, and phylogenetic diversity) in the compiled studies. Finally, we (v) identify which of the main drivers of biodiversity loss in tropical ecosystems (land cover change, climate change, fire dynamics, and exotic species) have been most studied in order to highlight the threats to biodiversity and, thus, contribute to the direction of conservation plans for tropical Savannahs.

## 2 Material and Methods

### 2.1 Data Collection and Screening

We use the Preferred Reporting Items for Systematic reviews and Meta‐Analyses (PRISMA) as a guideline for data collection and screening, which provides guidance for correctly reporting systematic scientific literature reviews (Page et al. 2021). We conducted our systematic search on the platform Web of Science using a combination of the following keywords in English: (bird) and (Savannah or Savannah), (mammal) and (Savannah or Savannah), (amphibian) and (Savannah or Savannah), (reptile) and (Savannah or Savannah). We opted for Web of Science because it is one of the platforms with the broadest coverage of scientific articles and presents a high overlap of search results with other platforms such as Scopus or Google Scholar (e.g., Mongeon and Paul‐Hus 2016; Pranckutė 2021). The searches were carried out considering the period from January 1945 to September 2025. We disregarded gray literature (monographs, dissertations, theses, reports, and technical reports), conference proceedings, and scientific notes. After searching, we obtained 3532 articles, of which 1560 were for birds, 1404 for mammals, 282 for reptiles, and 286 for amphibians.

### 2.2 Exclusion Criteria

**FIGURE 1:**
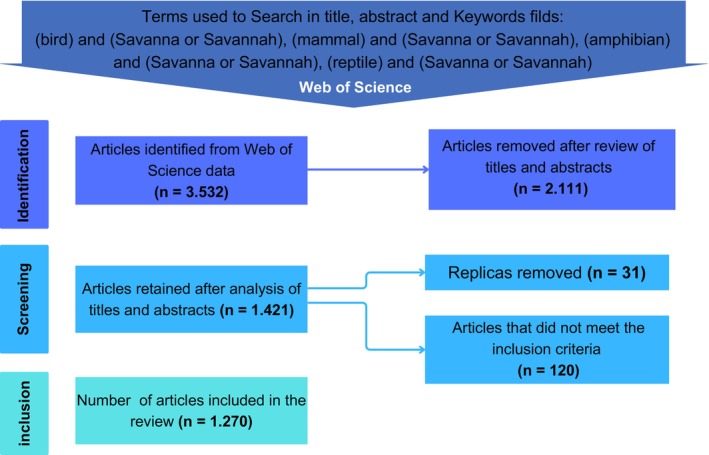
Workflow of the methodology used to perform the systematic literature review of research on vertebrate biodiversity in tropical savannahs.

**FIGURE 2:**
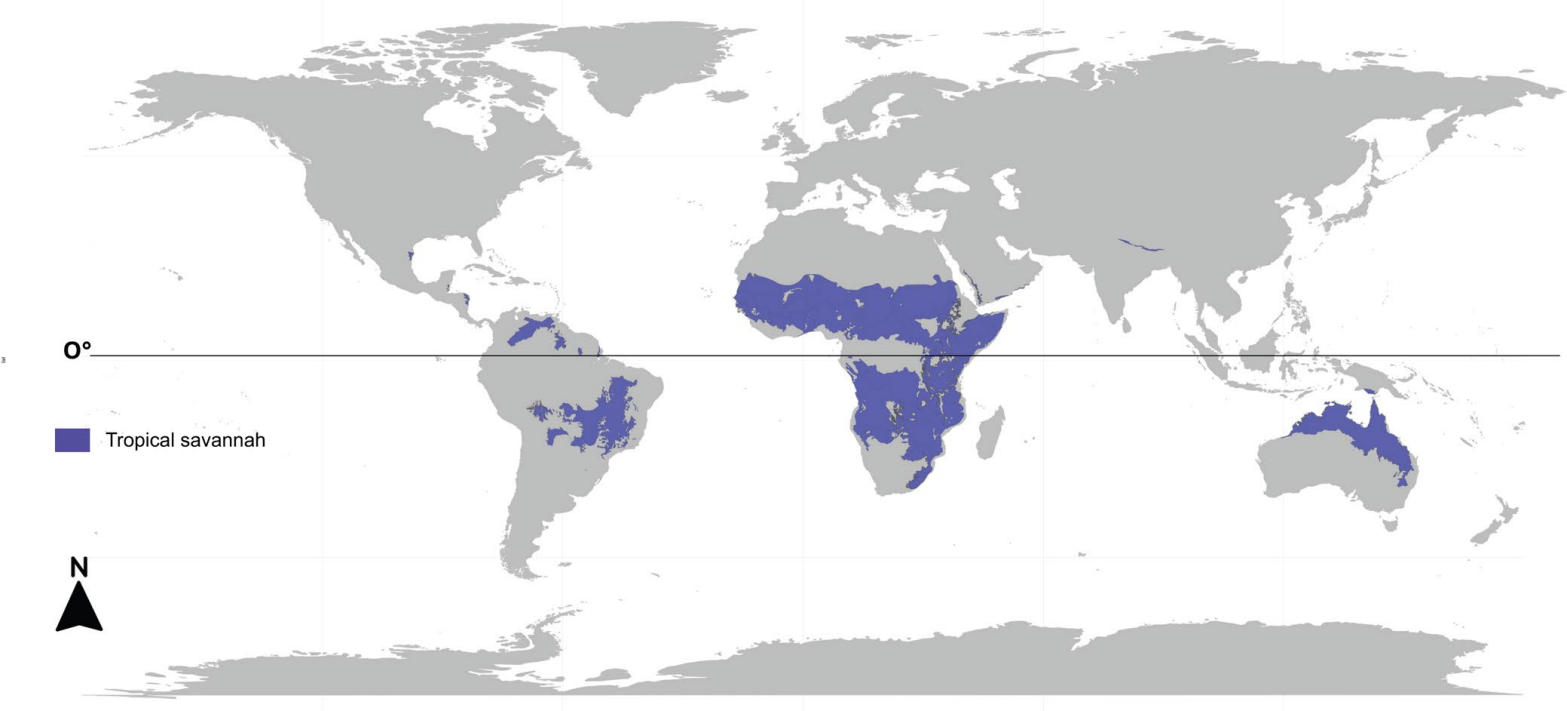
Global map showing the distribution of the savannah biome in the tropical region, modified from https://ecoregions.appspot.com.

Our systematic literature review of articles on vertebrate biodiversity in tropical Savannahs followed the flowchart in Figure 1. The selected articles were initially reviewed by reading the title, abstract, and keywords, and we excluded studies that did not include the taxonomic groups considered: amphibians, reptiles, birds, and mammals. After this selection, we excluded duplicates and performed a screening to exclude articles on research conducted outside tropical Savannahs (Figures 1 and 2); with a focus on paleontology or that did not present original research with primary and secondary data, which may include field studies, modelling inference, interviews, and laboratory studies that include genetic data (Figure 1).

### 2.3 Data Extraction and Analysis

We extracted the following information from each article: (1) year of publication, (2) journal title, (3) region of study (country and continent), and (4) taxonomic group (birds, mammals, reptiles, and amphibians). Additionally, for articles that had this focus, we extracted (5) biodiversity metrics (taxonomic, functional, and phylogenetic diversity), and (6) key drivers of biodiversity loss at tropical forest (land cover change, climate change, fire dynamics, and alien species—Gardner et al. 2010; Hogue and Breon 2022; Miranda et al. 2024; Morris 2010).

We explored this data using R version 4.4.1 (R Core Team 2020). For the data by region, we considered the total number of studies by region and country, as well as the density of studies, taking into account the coverage of Savannah biome by country. To estimate this density, we divided the total number of studies by the area of Savannah (in km^2^) that occurs within each country. Savannah cover was accessed from the global map of ecoregions (https://ecoregions.appspot.com/; Dinerstein et al. 2017; Figure 2) and, using QGIS Development Team (2022), we extracted the area in km^2^ of Savannah for each country. For the following countries, not covered by the ecoregion layers of Dinerstein et al. (2017), we sought information in the literature about the size of the Savannah area: French Guiana (Guyane 2017), Eswatini (Mucina et al. 2006), and Trinidad and Tobago (Comeau 1990). We also consider values of Human Development Index (HDI), available at Human Development Reports from United Nations Development Programme (UNDP) (https://hdr.undp.org/data‐center/human‐development‐index#/indicies/HDI) for each country except for French Guiana, which was not available in the UNDP report, being obtained through the Global Data Lab (GDL; https://globaldatalab.org/shdi/table/shdi/FRA/). HDI considers not only economic growth to assess a country's development, but also healthy life expectancy, longevity, and the population's level of education. To assess whether scientific production reflects the level of development of countries, we performed a Generalized Linear Mixed Model relating the density of studies as response variables to the HDI of each country. In these models, the continent was included as a random variable.

## 3 Results and Discussion

### 3.1 How Many Studies Focus on Amphibians, Reptiles, Birds, and Mammals in Tropical Savannahs?

**FIGURE 3:**
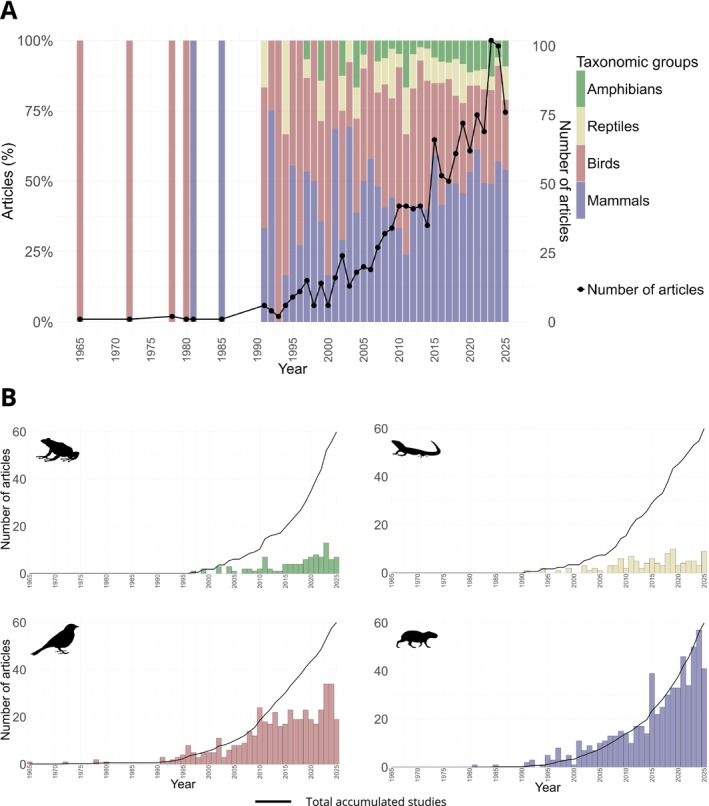
A temporal trend in scientific publications on vertebrates in tropical savannahs between 1965 and 2025. (A) Total number of accumulated articles (black line) and percentage of studies (colored bars) at each year and by vertebrate group in tropical savannahs, (B) total number of accumulated articles (black line) and number of articles published (bars) at each year and for each group vertebrate group in tropical savannahs (B).

Our searches returned a total of 3532 articles, of which 2111 were excluded after review of titles and abstracts for not presenting research on the focal taxonomic groups of the review, 31 were excluded for being duplicates and 120 were excluded for not meeting the other inclusion criteria (Figure 1). Thus, 1270 published articles investigating terrestrial vertebrates of Savannahs were included in our systematic review (Figure 1). Of these 1270 articles, 96 investigated more than one taxonomic group simultaneously (e.g., Ogada et al. 2008; Price et al. 2010; Silveira et al. 2020; Mukomberanwa and Ngorima 2025). The distribution of articles for taxonomic groups had the respective values: mammals (*n* = 608, 48%), birds (*n* = 462, 36%), reptiles (*n* = 102, 8%), and amphibians (*n* = 98, 8%; Figure 3A). Although the number of published articles has increased over the last 30 years (Figure 3B), this trend for the taxonomic groups remains (Figure 3B).

While studies of birds and mammals are invaluable for conservation, increasing the focus of research on other taxonomic groups can be crucial for the conservation of biodiversity, especially when we consider the increasing number of threatened or endangered species for both groups (Cox et al. 2022; Luedtke et al. 2023). Currently, 41% of amphibians and 21% of reptiles that occur in the world are at risk of extinction (Cox et al. 2022; Stuart et al. 2004; Whiles et al. 2006; Böhm et al. 2013; Harfoot et al. 2021; IUCN 2024), and amphibian populations are declining at much greater rates than birds and mammals (Stuart et al. 2004; Whiles et al. 2006). Furthermore, currently 1502 species of reptiles (46.29%), 908 species of amphibians (27.98%), 797 species of mammals (24.56%), and 38 (1.17%) species of birds are categorized as Data Deficient on the IUCN Red List, meaning there is currently insufficient data to assess their conservation status, highlighting the urgent need for more studies particularly of reptiles and amphibians (Bland et al. 2015, 2017; Hochkirch et al. 2021; Roberts et al. 2016).

### 3.2 Where Are the Geographic Gaps in Scientific Knowledge on Tropical Savannah Vertebrates?

**FIGURE 4:**
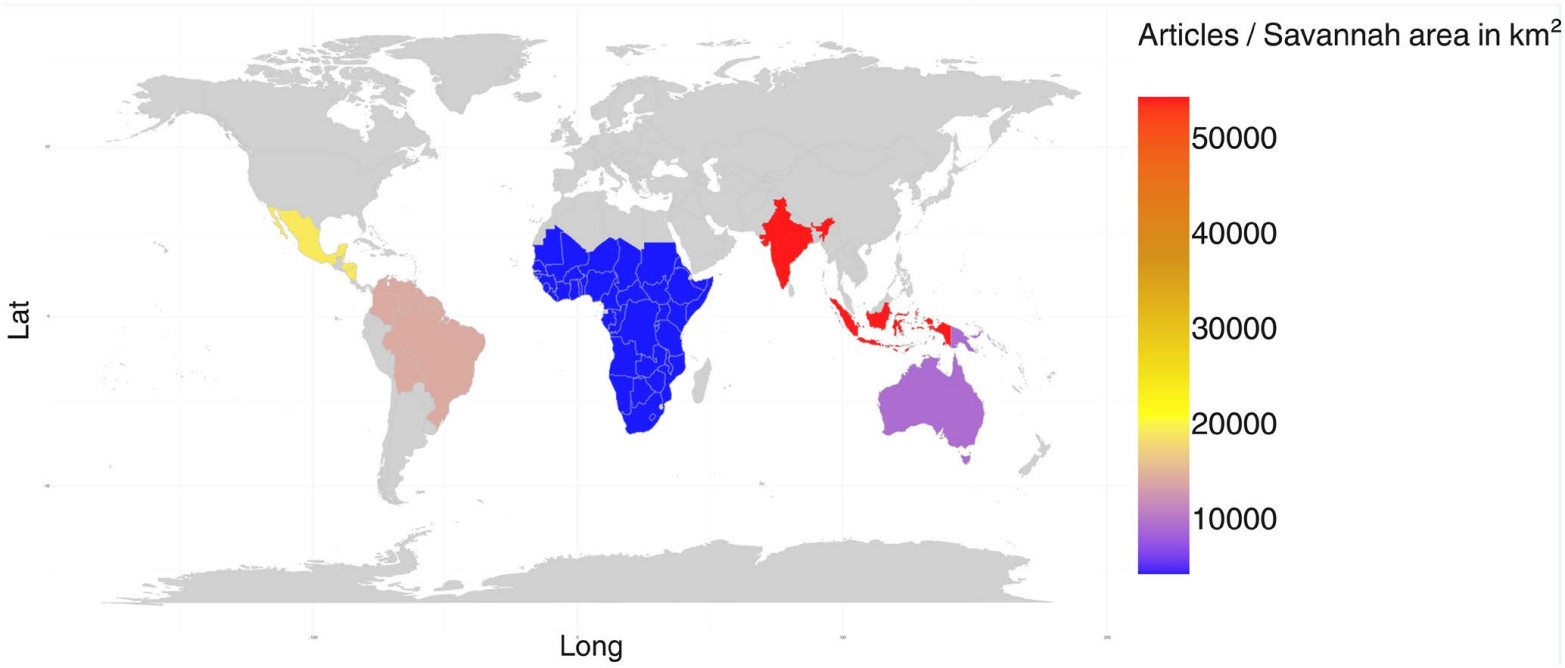
Geographical distribution of the number of studies by savannah area (in km^2^) that occurs within each continent. Density values were raised to the sixth power (1×10^6^) for better visualization.

**FIGURE 5:**
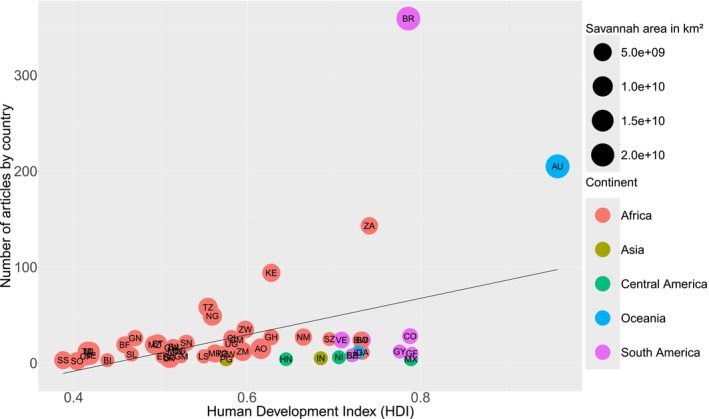
Number of published studies in relation to the human development index of countries in the tropical savanna region. Country codes used are: Angola (AO), Australia (AU), Belize (BZ), Benin (BJ), Bolivia (BO), Burundi (BI), Burkina Faso (BF), Brazil (BR), Central African Republic (CF), Congo (CG), Ivory Coast (CI), Democratic Republic of the Congo (CD), Colombia (CO), Cameroon (CM), Eritrea (ER), Ethiopia (ET), French Guiana (GF), Ghana (GH), Gabon (GA), Guyana (GY), Gambia (GM), Guinea (GN), Guinea‐Bissau (GW), Honduras (HN), Indonesia (ID), India (IN), Liberia (LR), Lesotho (LS), Kenya (KE), Malawi (MW), Mexico (MX), Mauritania (MR), Mali (ML), Niger (NE), Nigeria (NG), Nicaragua (NI), Namibia (NM), Papua New Guinea (PG), Rwanda (RW), Sudan (SD), Sierra Leone (SL), Senegal (SN), Somalia (SO), Suriname (SR), South Sudan (SS), Eswatini (SZ), Tanzania (TZ), Chad (TD), Togo (TG), Uganda (UG), South Africa (ZA), Zambia (ZM), Zimbabwe (ZW), and Venezuela (VE). The density of studies was raised to the ninth power (1×10^9^) for better visualisation.

For all vertebrate groups, the geographic regions that concentrated the largest number of studies were Africa (*n* = 875, 55%), South America (*n* = 455, 29%), and Oceania (*n* = 209, 13%), followed by Asia (*n* = 16, 1%), Central America (18, 1%), and North America (*n* = 4, 0.1%). However, considering the density of studies per Savannah area, our results show that Africa has a lower proportion of scientific research compared to Oceania and South America, respectively (Figure 4). This result has been found in other global‐scale studies that consider different regions and biomes (e.g., Trimble and van Aarde 2012; Titley et al. 2017; Xavier et al. 2023; Caldwell et al. 2024). Furthermore, the geographic bias that our data shows for the distribution of studies in Savannahs was positively related to the HDI of countries (*R* ^2^ = 0.20, *p* = 0.0001; Figure 5). Global systematic reviews that jointly evaluated the different global climate zones and incorporated different vertebrate groups showed a positive relationship between the number of published studies and the Gross Domestic Product (GDP). This index measures a country's growth rate through the value of its Gross Domestic Product, with Africa concentrating most countries with the lowest GDPs (Titley et al. 2017). This suggests that the level of education, quality of life, and GDP per capita influence scientific production on vertebrates not only in tropical Savannahs, but throughout the world.

**FIGURE 6:**
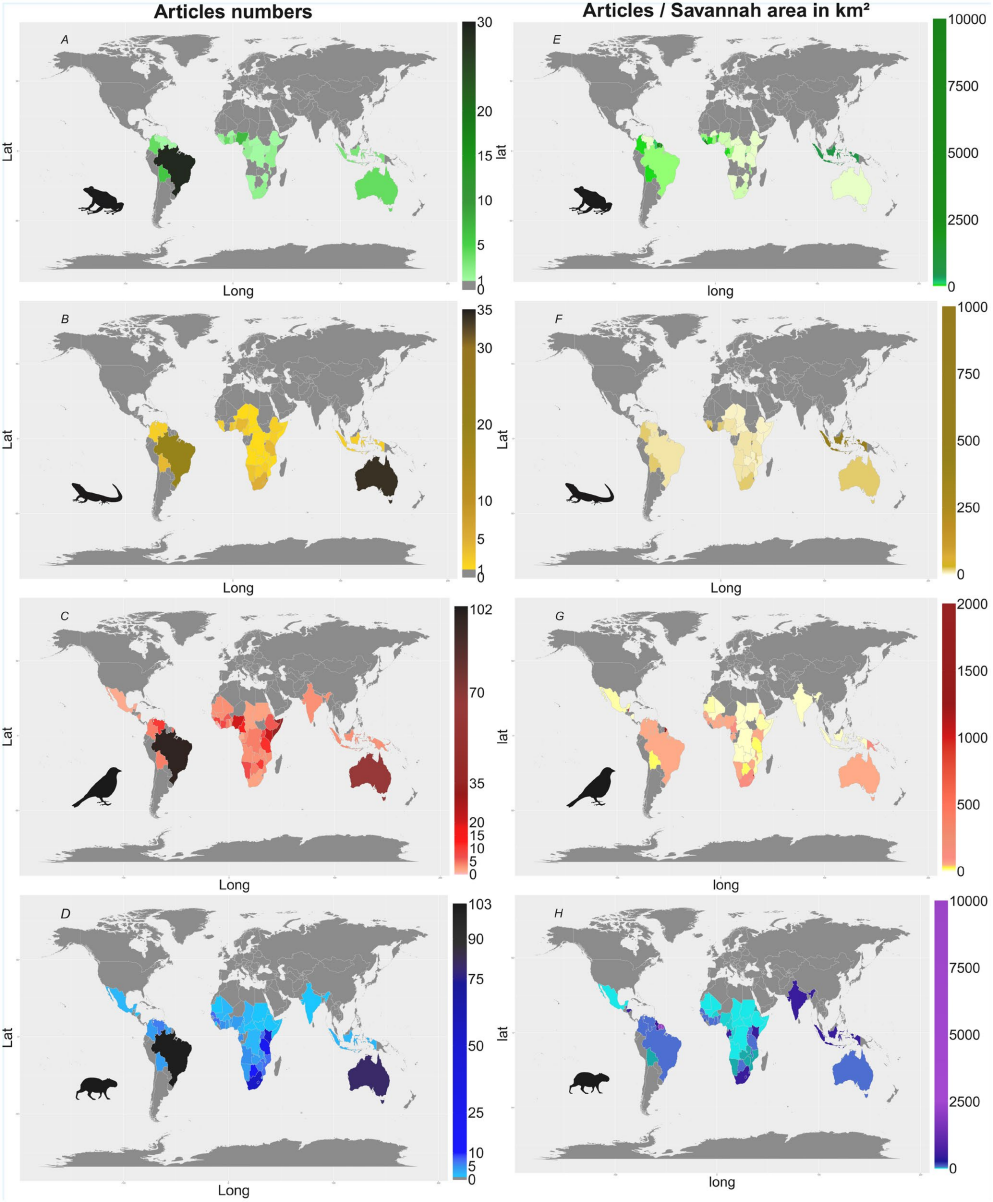
Geographical distribution of published studies on vertebrate biodiversity in tropical savannahs found on the Web of Science platform. Figure (A) amphibians, (B) reptiles, (C) birds, and (D) mammals represent the number of studies found in each country for the respective taxonomic groups. Figure (E) amphibians, (F) reptiles, (G) birds, and (H) mammals represent the density of studies in relation to the savannah area (in m^2^) of each country.

The low density of studies in Africa demonstrated by our results may have been driven by low scientific output in Central African countries such as Chad, the Central African Republic, and the Democratic Republic of Congo; West African countries such as Mali and Niger; and East African countries such as Sudan and South Sudan, which presented a lower number and density of articles for all vertebrate groups (Figure 6). In contrast, our results also demonstrated that countries such as Ghana in the West, and South Africa, Tanzania, and Kenya in the South/Southeast of the continent, have numbers and densities of publications comparable to those of South America and Oceania (Figure 6). The differences shown by our study regarding the number of studies between Central African countries and those in Southern and Southeastern Africa can be explained by a temporal bias and sampling effort toward the areas richer in biodiversity on the continent (Farooq et al. 2021). Furthermore, sites that have already been sampled reduce logistics costs and therefore facilitate access for new studies (Reddy and Dávalos 2003; Farooq et al. 2021). Economic factors may also explain this difference between regions of the African continent. Countries such as South Africa, Tanzania, and Kenya have large local economies and emerging markets and, therefore, can direct more resources to scientific research (FMI 2024; Hanlin and Tigabu 2017). Internal conflicts may be another factor contributing to this discrepancy between African countries. Civil wars in many Central African countries, such as Angola, Chad, Mali, Mozambique, Niger, the Democratic Republic of Congo, the Central African Republic, Sudan, and South Sudan, have damaged their economies, their natural environments, and the free movement of researchers, reducing scientific output in these regions (Nackoney et al. 2014; Daskin et al. 2016; Dudley et al. 2002). Finally, internal conflicts, such as civil war, have led to massive poverty among the population, high mortality rates, low life expectancy, and a precarious education system (Kansal and Cole 2019), which in turn place Chad, Mali, Niger, the Central African Republic, Sudan, and South Sudan among the 10 countries with the lowest HDI in the world (UNDP 2024).

South America and Oceania presented a contrasting pattern in terms of the number and density of articles per country (Figure 6). Countries with large geographic areas, such as Australia and Brazil, whose Savannah areas are the largest among the countries analyzed (2,354,286.493 and 2,088,093.75 km^2^, respectively—Supporting Information S1), had the largest number of studies (Figure 6). However, the density of studies from these countries was lower compared to a small territory like French Guiana (251 km^2^; Stier et al. 2020), for example, regardless of the taxonomic group analyzed (Figure 6). Brazil and Australia are countries of continental proportions and research and funding efforts need to be proportionally large to cover the extent of these areas. Therefore, it is necessary to increase efforts to fill the gaps, mainly due to the intense anthropogenic disturbances that these countries have suffered in recent decades, such as forest fires (Boer et al. 2020; Cardil et al. 2020) and conversion of natural areas to agricultural systems (Sano et al. 2010; Aragão et al. 2022).

### 3.3 Are We Looking at All Dimensions of Tropical Savannahs' Biodiversity?

Of the 1270 articles included in this study, 567 focused on biodiversity metrics. Taxonomic diversity was the most used metric in the studies (*n* = 527, 93%), with low use of functional (*n* = 25, 4%) and phylogenetic (*n* = 15, 3%) diversity. Only 27 articles (5%) used more than one diversity metric, of which 14 articles (3%) measured two diversity metrics simultaneously and 11 articles (2%) used all three diversity metrics.

**FIGURE 7:**
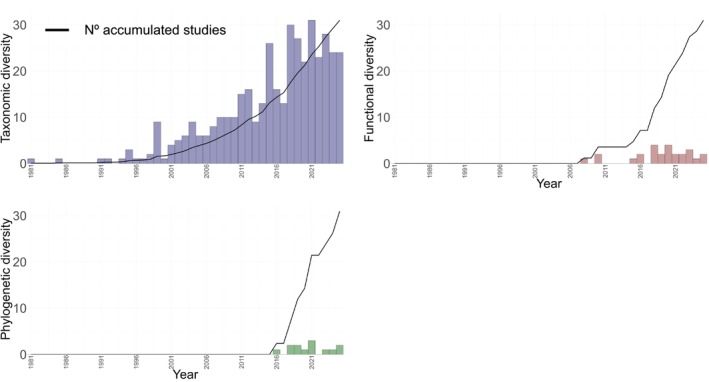
A temporal trend in scientific publications that measured each dimension of taxonomic, functional, and phylogenetic diversity on vertebrate biodiversity in tropical savannahs.

The low number of studies on functional and phylogenetic diversity can be explained by the recent development of metrics for these dimensions of biodiversity (Faith 1992; Petchey and Gaston 2002; Villéger et al. 2008; Kembel et al. 2010; Villéger et al. 2010), while taxonomic metrics are older and more widely used (Shannon 1948; Simpson 1949; Xavier et al. 2023). In the case of tropical savannahs, functional diversity only began to be used in 2008 and phylogenetic diversity in 2016, while taxonomic diversity has been used since 1980 and has shown an exponential increase after the 2000s (Figure 7). Despite advances in computational tools to  infer these dimensions (Mammola et al. 2021; Palacio et al. 2022), the lack of data, functional characters, and phylogenies that cover all species makes it difficult to incorporate functional and phylogenetic diversity metrics into research (Diniz‐Filho et al. 2013; Etard et al. 2020; Hortal et al. 2015).

Biodiversity can be considered multidimensional, including taxonomic, functional, and phylogenetic dimensions (Faith 1992; Petchey and Gaston 2002; Shannon 1948; Simpson 1949). In a world undergoing intense changes caused by habitat loss and alteration, biological invasions, and climate change, it may be necessary to increase the use of multidimensional approaches to better understand how biodiversity will adapt to these changes. For example, habitat degradation may lead to increases in taxonomic diversity as a result of increases in generalist or opportunistic species while simultaneously causing functional redundancy and loss of species with unique characteristics linked to habitat quality and maintenance (Basile 2022; Leitão et al. 2016; Naeem et al. 2012), for example, via seed dispersal (Westcott et al. 2005). In terms of phylogenetic diversity, regional‐scale processes, where invasions outnumber extinctions of phylogenetically unique species from different localities, can lead to an increase in taxonomic diversity and phylogenetic homogenization, because biological invaders often belong to species‐rich families and tend to be closely related to native species (Winter et al. 2009; Pyšek 1998). Furthermore, more phylogenetically diverse communities are more resilient to environmental changes (Forest et al. 2007). Therefore, increasing research efforts to assess the functional and phylogenetic dimensions of biodiversity in tropical Savannahs in relation to major biodiversity threats is essential to promote conservation plans that enhance ecosystem resilience to disturbance.

### 3.4 Scientific Gaps and Trends About the Main Drivers of Biodiversity Loss Relevant to the Conservation of Tropical Savannahs

**FIGURE 8:**
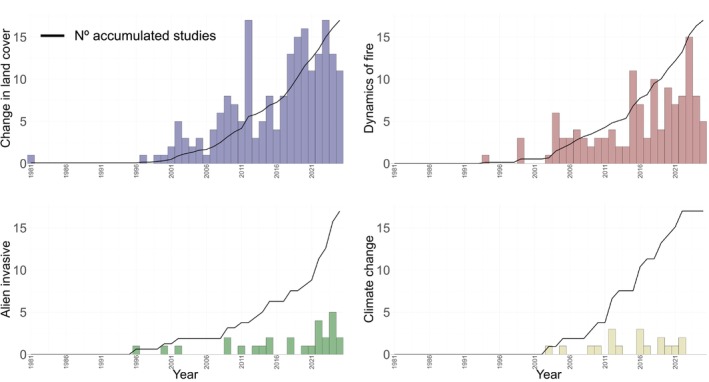
A temporal trend in scientific publications on the main drivers of vertebrate biodiversity loss in tropical savannahs.

Of the 1270 articles included in our analysis, 380 studies included analysis of the effects of the main drivers of global biodiversity loss. Our results showed an increase in the frequency of studies across all drivers of biodiversity loss analyzed since the early 1990s. Studies investigating the effects of fire dynamics (*n* = 179, 47%) and land use changes (*n* = 163, 42%; Figure 8) had the highest number of published articles. Furthermore, we observed a 175% increase in the publication of articles investigating land use change and 75% for fire dynamics after 2007 (Figure 8). Invasive species and climate change contributed 25 (6%) and 16 (5%) articles, respectively.

**FIGURE 9:**
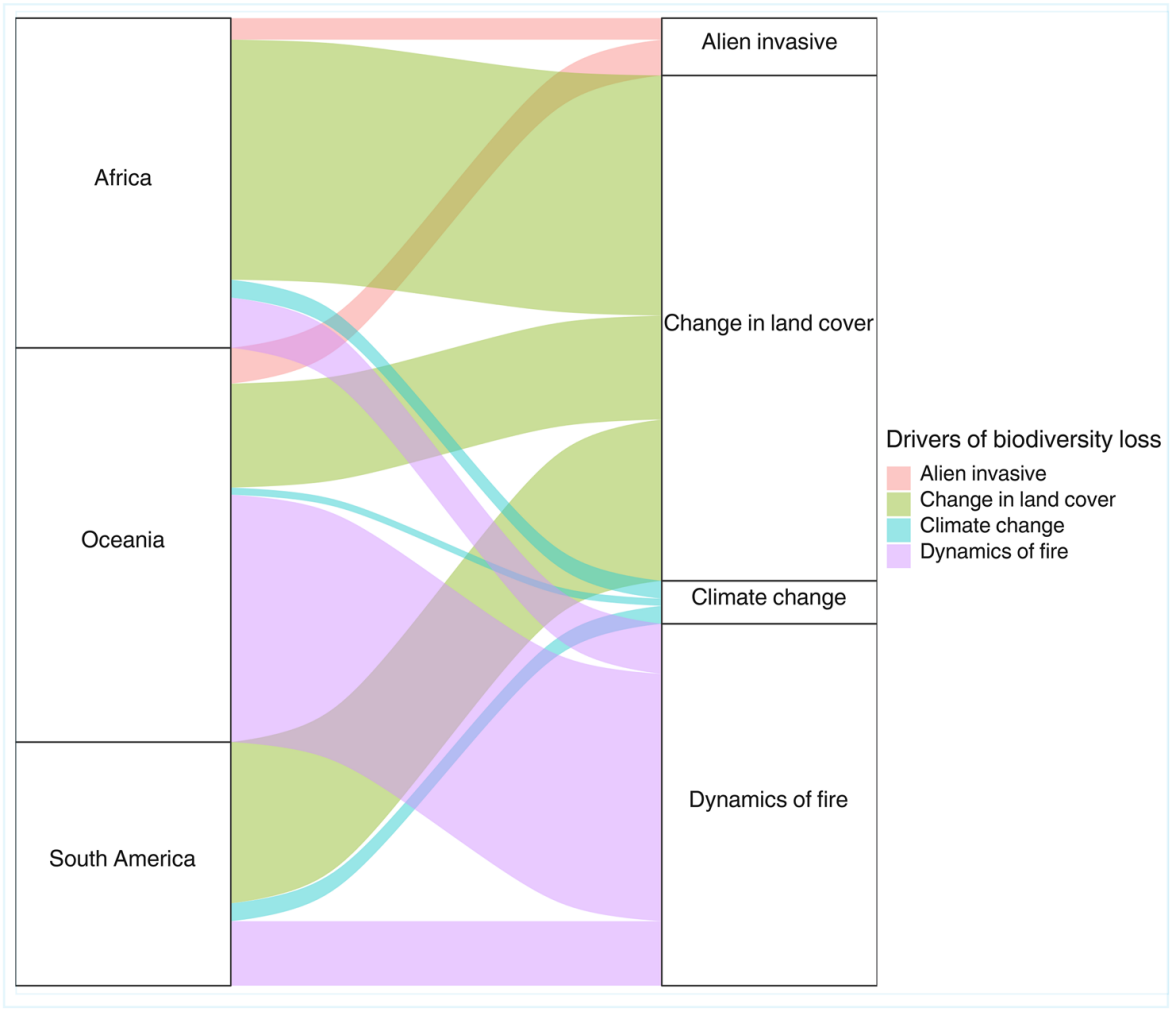
Number of articles focused on the main drivers of vertebrate biodiversity loss in relation to the continents that encompass tropical savannahs.

The geographic distribution showed a greater number of articles related to land cover change in Africa (*n* = 87, 53%) and South America (*n* = 45, 27%; Figure 9). For wildfires, we found that Oceania, particularly Australia, holds the highest number of published articles (*n* = 84, 46%; Figure 9). Regarding invasive species, our search found only 25 articles distributed between Oceania (*n* = 16, 64%), Africa (*n* = 7, 28%), and South America (*n* = 2, 7%; Figure 9). For climate change, the studies were distributed between Africa (*n* = 8, 50%), South America (*n* = 5, 31%), and Oceania (*n* = 3, 19%; Figure 9).

The increased frequency of studies related to the main drivers of biodiversity loss after the 1990s may be related to the worsening and intensification of climate change, land use, forest fires, and the invasion of invasive species (Geist and Lambin 2002; Georgina 2014; FAO 2022; IUCN 2024; Jolly et al. 2015; Murphy and Russell‐Smith 2010). Globally, 58.4% of the Earth's surface has already been transformed by human activities, and tropical Savannahs are among the biomes that have experienced the greatest loss of natural cover, with 13.3% (655,000 km^2^) of their area converted (Williams et al. 2020). That way, the greater effort expended in scientific research on the consequences of changes in land use cover was expected, as it is directly linked to the loss and extinction of species and is a priority area for study for the conservation of species and ecosystems (Stefanes et al. 2018). Land use change, except for Oceania, was the most studied driver of biodiversity loss, with Africa presenting the largest number of studies. Agricultural intensification that has occurred in recent decades has converted large areas of native Savannahs into pastures and commercial plantations on the African continent (Bullock et al. 2021; FAO 2022; Geist and Lambin 2002; Niklaus et al. 2015; Wingate et al. 2016). Furthermore, population growth rates in East and Sub‐Saharan Africa are among the highest in the world (UN 2024), generating high demands for wood (Sulaiman and Abdul‐Rahim 2020), particularly for energy generation (FAO 2022).

Land cover change is a key driver of biological invasion. An estimated 21.7% of native species extinctions are driven by the interaction between agriculture and invasive species (Blackburn et al. 2019; Harfoot et al. 2021). Globally, invasive species represent one of the main threats to native species (IPBES 2019; IUCN 2024), due to predation, competition, and disease transmission (Byers 2002; Frank et al. 2014; Lips 2016; Van Wilgen et al. 2022). In addition, they can alter abiotic characteristics and ecosystem functioning (Ehrenfeld 2003), increasing pressure on native species. However, our review indicated that there are few studies on this topic for vertebrate diversity of the tropical Savannahs, which is a gap that needs to be filled by the scientific community. This is especially urgent in regions such as Africa and South America, where large conversions of landscapes to agriculture are occurring (Carvalho et al. 2019; Duan and Tan 2019; Bullock et al. 2021).

Climate change is a major cause of species extinction (Maclean et al. 2011; Thomas et al. 2004; IUCN 2024; Montràs‐Janer et al. 2024). However, only 6% (*n* = 16) of studies focused on the effects of climate change on biodiversity (Figure 9), with birds (*n* = 7), mammals (*n* = 8), and reptiles (*n* = 1). The small number of studies on climate change may reflect a temporal bias, as studies on the effects of climate change on biodiversity only began to become more frequent from 2010 onward (Farooq et al. 2021), a result also found here for tropical savannahs (Figure 8).

One of the main impacts of climate change on biodiversity is the increase in the intensity and frequency of wildfires (Cunningham et al. 2024; Jolly et al. 2015). In our study, we found that Oceania, and particularly Australia, holds the highest number of published articles related to fire dynamics (Figure 9). Natural fire has been a fundamental element for the formation and maintenance of Australia's Savannah ecosystems for millions of years (Crisp et al. 2011). Aboriginal peoples control low‐intensity fires in small areas limited to the grass layer of the ground for subsistence hunting of small animals, which also promotes the heterogeneity of vegetation and the diversification of associated fauna (Bliege Bird et al. 2008; Bird et al. 2024; Trauernicht et al. 2015; Wurster et al. 2021). However, with the arrival of European settlers in Australia in the late 18th century, anthropogenic influences on fire activity became more pronounced due to large‐scale agricultural processes (Bowman et al. 2020; Paterson 2018). The combined effects of colonization and climate change have accelerated the decline of many taxa in Australian Savannahs (Murphy and Russell‐Smith 2010; Teunissen et al. 2023; Ziembicki et al. 2015).

Due to this susceptibility and potential risks of wildfires in Australia, researchers from these regions may have focused their efforts on understanding the relationship between fire dynamics and biodiversity to combat or prevent future threats to biodiversity. Another factor that may contribute to the greater number of studies in Oceania is the fact that the continent is one of the richest and most developed in the tropical Savannah belt, especially Australia, which guarantees greater financial and technical support to research institutions in the region. In addition, Australia is among the 20 most megadiverse countries in the world, which in turn becomes one of the critical points to be prioritized for biodiversity conservation (Myers et al. 2000). However, this result is not reflected in Brazil, despite the increased number of fires in recent years, mainly in non‐forest biomes such as the Pantanal and Cerrado, causing the loss of around 17 million vertebrates in a single year (Tomas et al. 2021). Even with the increase in fire outbreaks, there are few studies on the impacts on biodiversity in Brazil (e.g., Durigan et al. 2020; Mendonça et al. 2015; Prada and Marinho‐Filho 2004).

## 4 Conclusions

Our results show four biases in scientific studies on tropical Savannah vertebrates (I) taxonomic bias, with mammals and birds being the most studied vertebrates; (II) geographic bias in the distribution of studies, with the density of studies per area of an ecosystem being closely related to the Human Development Index (HDI) of each country, with Africa concentrating the majority of countries with the lowest HDI and a lower number and density of studies; (III) bias regarding the use of different dimensions to quantify biodiversity, with most studies focusing only on taxonomic diversity; and (IV) bias toward the main drivers of biodiversity loss, with the topics that have received the least attention in the literature being those related to invasive species and climate change.

Gaps in global data on vertebrate groups can negatively impact the conservation of tropical Savannah biodiversity. The smaller number of studies for certain taxonomic groups (e.g., amphibians and reptiles) or some areas of the globe (e.g., Central Africa) can generate erroneous estimates of biodiversity and underestimate areas for targeting conservation and preservation actions. Ensuring a broader knowledge of biodiversity can guide decision‐makers to achieve the goals proposed by collaborative agreements such as the Kunming‐Montreal Global Biodiversity Framework (GBF 2022), which aims to preserve and restore ecosystems, minimize the impacts of invasive species, and reduce biodiversity loss.

Since our approach focused on searching for studies that explicitly mention the term “savanna or savannah,” it is possible that this approach favored the inclusion of research conducted at local and regional scales, where the characterization of the biome is explicitly defined in the publications. Therefore, studies on global modelling scales, macro‐ecological approaches, or investigations encompassing multiple biomes may not have been retained if they did not explicitly mention the term “savanna or savannah” in the description of the study area, title, abstract, or keywords. Therefore, the global literature on modeling, macroecology, and investigations encompassing multiple biomes in the face of climate change may be underrepresented in this synthesis.

## 5 Policy Implications for Conservation

Currently, public and private finance flows for nature‐based solutions to address climate change, biodiversity loss, and ecosystem degradation amount to just US$200 billion annually (UNEP 2022). However, to meet the Rio Convention targets to limit climate change to 1.5°C, as well as the Global Biodiversity Framework target of preserving 30% of land and sea by 2030 and achieving land degradation neutrality, finance flows need to triple by 2030 and quadruple by 2050 (UNEP 2023). In contrast, around 7% of global GDP, or US$7 trillion, is invested annually in activities such as extensive agriculture, fossil fuels, forestry and logging, which negatively impact biodiversity (UNEP 2023). However, the agricultural sector rarely takes responsibility for the environmental liabilities related to soil depletion and biodiversity loss. Therefore, it is necessary to realign financial flows that negatively impact nature, allocating part of these resources to activities that aim to curb biodiversity loss and promote ecosystem resilience, to provide the ecosystem services on which people depend so much. It is essential to increase research subsidies to expand scientific knowledge about the leading causes of biodiversity loss and habitat degradation to help create projects focused on the conservation of Savannah and other non‐forest ecosystems. Since tropical Savannahs are the most prone to conversion to agriculture and pasture, increasing funding for biodiversity research in these areas is urgent. Another effective measure would be the expansion of the Soy Moratorium to the Cerrado and Amazonian Savannahs, given its effectiveness in the Brazilian Amazon (Nepstad et al. 2014; Overbeck et al. 2015; Carvalho et al. 2019; Câmara dos Deputados 2024). Finally, researchers should collaborate to support research in key geographic regions, especially in Central Africa, to fill the gaps in the study of vertebrates in tropical Savannahs. To this end, it is crucial that African Union (AU) member states meet their target of investing 1% of GDP in biodiversity research and conservation each year (African Wildlife Foundation 2025). In addition to funding, more educational innovation and initiatives for developing technical capacities for future generations are needed (Bezeng et al. 2025). Therefore, it is essential to create more national programs like the National Research Foundation (NRF) of South Africa in other regions of Africa, whose goal is to create instruments to finance research projects and promote scientific career development. More funding is also needed for African universities, as well as adequate funding for African academics to prevent brain drain. Furthermore, more initiatives such as citizen science, technical capacity building, and the inclusion of indigenous peoples and traditional communities can accelerate the collection of biodiversity information. However, any funding sources or collaborations that come from foreign countries, especially when they are Global North–Global South collaborations, must pay attention to issues of recognizing the contributions of all parties involved, and equitable participation and distribution of costs and benefits of the research, in order to avoid “helicopter science” and other neocolonial practices.

## Author Contributions

**Marcelo Martins Ferreira:** conceptualization (equal), data curation (equal), formal analysis (equal), investigation (equal), methodology (equal), project administration (equal), resources (equal), writing – original draft (equal). **Karen Mustin:** methodology (equal), writing – review and editing (equal). **Paulo Estefano Dineli Bobrowiec:** conceptualization (equal), data curation (equal), funding acquisition (equal), investigation (equal), methodology (equal), project administration (equal), resources (equal), supervision (equal), writing – review and editing (equal). **William Douglas Carvalho:** conceptualization (equal), data curation (equal), funding acquisition (equal), investigation (equal), methodology (equal), project administration (equal), resources (equal), supervision (equal), writing – review and editing (equal).

## Funding

This work was supported by the Fundação de Amparo à Pesquisa do Estado do Amazonas (01.02.016301.005462/2024‐32); Coordenação de Aperfeiçoamento de Pessoal de Nível Superior (88881.934045/2024‐01); Ministerio de Ciencia, Innovación y Universidades (BG22/00121, CA3/RSUE/2021‐00197, and RYC2023‐045231‐I); and the National Geographic Society (NGS‐96963R‐22).

## Conflicts of Interest

The authors declare no conflicts of interest.

## Supporting information


**Appendix S1:** ece372917‐sup‐0001‐AppendixS1.csv.

**Appendix S2:** ece372917‐sup‐0002‐AppendixS2.xlsx.

## Data Availability

All the data and materials generated or analyzed during this study are included in this published article and its Supporting Information files (see Supporting Information S1 and S2).
